# EBV-Associated Smooth Muscle Neoplasms: Solid Tumors Arising in the Presence of Immunosuppression and Autoimmune Diseases

**DOI:** 10.1155/2008/859407

**Published:** 2008-11-30

**Authors:** Kimberly Moore Dalal, Cristina R. Antonescu, Ronald P. DeMatteo, Robert G. Maki

**Affiliations:** ^1^Department of Surgery, Memorial Sloan-Kettering Cancer Center, New York, NY 10065, USA; ^2^Department of Surgery, David Grant United States Air Force Medical Center, Travis Air Force Base, CA 94535, USA; ^3^Department of Surgery, University of California at San Francisco, CA 94143, USA; ^4^Department of Pathology, Memorial Sloan-Kettering Cancer Center, New York, NY 10065, USA; ^5^Department of Medicine, Memorial Sloan-Kettering Cancer Center, New York, NY 10065, USA

## Abstract

*Background*. Epstein-Barr virus (EBV)-related smooth muscle neoplasms (SMNs) have been associated with immune dysregulation, most notably in patients who have undergone solid organ transplantation or in patients with HIV/AIDS. *Objective*. to report our experience with EBV-related neoplasms as well as describing the first EBV-related SMN in the setting of administration of glucocorticoids and the tumor necrosis factor inhibitor etanercept. *Design*. We have case reports, of minimum 3-year follow-up, 2002–2005. *Setting*. It was held in an academic and tertiary referral cancer center. *Patients*. Patients are with dysregulated immunity after solid organ transplantation, HIV/AIDS, or with psoriasis after treatment with etanercept. *Interventions*. There were discontinuation of etanercept, right hepatic trisegmentectomy, and chemotherapy. *Measurements*. We use survival as a measurement here. *Results*. Patients who were able to withstand reduction in immunosuppression survived. Surgical resection or chemotherapy was successful in delaying progression of disease. *Limitations*. There was a relatively short follow-up for these slow-growing neoplasms. *Conclusion*. EBV-related SMNs have variable aggressiveness. While chemotherapy may slow disease progression, resection and improving the host immune status provide the best opportunity for primary tumor control.

## 1. INTRODUCTION

Immunocompromised patients have an increased incidence of immunosuppression-associated malignancies. While unusual, smooth muscle neoplasms (SMNs), such as leiomyomas and leiomyosarcomas, occur at a greater frequency in immune-dysregulated patients compared to the general population [1, 2]. SMNs have been reported in immunodeficient patients after solid organ transplants [2–5], with acquired immune deficiency syndrome (AIDS) [6], or with congenital conditions [7–10]. SMNs are strongly associated with Epstein-Barr virus (EBV) [4, 6, 9].

We report three cases of EBV-associated SMN, one in a patient with psoriasis who had received the tumor necrosis factor (TNF) antagonist etanercept, and describe our institution’s recent experience with this entity.

*Patient* 1. A 55-year-old Asian man presented in 2002 with hepatitis B, hypertension, penile carcinoma status postresection and lymphadenectomy in 1988, and chronic renal insufficiency. He had undergone cadaveric renal transplant in 1994 and remained rejection free on cyclosporine, azathioprine, mycophenolate mofetil, and prednisone.

In March 2002, he developed abdominal pain and a 15-pound weight loss. Computed tomography (CT) scans (7/02) revealed an unresectable 14 cm caudate-based mass; multiple pulmonary nodules were visualized. Alpha-fetoprotein level was normal. Core needle biopsy demonstrated a well-differentiated SMN. His immunosuppressive therapy was not reduced because of the likelihood of transplant rejection. The patient refused systemic chemotherapy. By November 2002, he developed jaundice and increasing debilitation. CT scan revealed rapid disease progression with pulmonary metastases and ascites. He died later that month.

*Patient* 2. An 18-year-old woman diagnosed with congenital AIDS began to experience abdominal pain at age 11, in 1995. Colonoscopy was initially negative, but laparoscopy in 1997 revealed a well-differentiated SMN arising from the rectosigmoid, unchanged in 1998 and 1999 on subsequent colonoscopies. In October 2002, during an evaluation for persistent abdominal pain and areflexia in the left ankle and knee, magnetic resonance imaging (MRI) revealed multiple abdominal and paraspinal masses. She underwent resection of the lumbar paraspinal tumor and rod placement. Pathology demonstrated an EBV-related SMN.

By 2003, CD4 count was 8, and viral load was 15 000. Medications included stavudine and lamivudine. She then presented, wheel-chair bound, with fatigue and refractory abdominal pain, weighing 19 kg. Eastern Cooperative Oncology Group (ECOG) performance status was 4. CT scans (3/03) demonstrated low-attenuation lesions in the liver and a 9 cm mass arising between the stomach and the liver. She demonstrated marked clinical improvement after starting doxorubicin in October 2003, and 5–10% decrease in masses on reimaging. She interrupted therapy in mid 2004, starting dacarbazine in November 2004 for persistent disease affecting the chest, abdomen, and paraspinal areas. Despite her noncompliance and neutropenia, the tumors decreased in size further, her weight doubled, she began to walk, and has an ECOG performance status of 1 as of January 2006. She is still alive with disease (AWD) but without progression as of last follow-up in October 2008.

**Figure 1:**
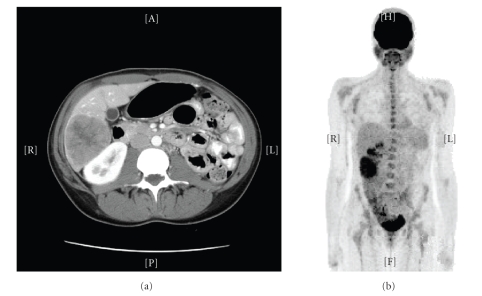
CT and PET scans of EBV-related SMN of liver. CT scan of the abdomen and pelvis (8/05) from Patient 3 revealed multiple masses, mainly in the right lobe and involving segment 4. The largest mass measured 6.8 × 5.8 cm and was located in the right inferior lobe (a). FDG-PET (9/05) demonstrated areas of hypermetabolism in the inferior aspect of the right hepatic lobe (SUV max 5.7), and two other neighboring foci (SUV max 5) (b).

**Figure 2:**
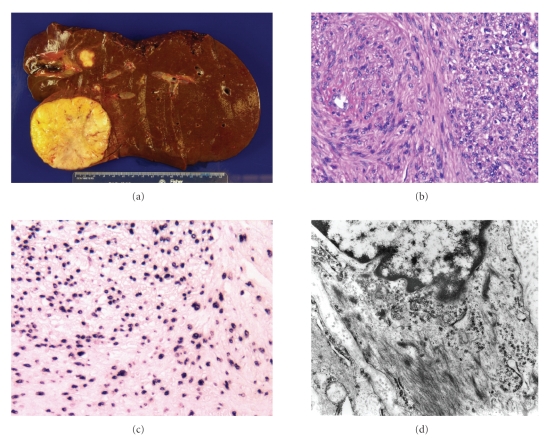
Gross and microscopic findings from EBV-related SMN of liver. Liver specimen from Patient 3 measured 17 × 13.2 × 7.6 cm. Four nodules were found, ranging in size from 0.4 cm to 6.7 cm (a). Histologic features on H&E staining included moderate increased cellularity (with areas containing rounder, less fusiform cells), lack of nuclear pleomorphism, and a low mitotic rate (1 mitotic figure/50 high-powered field) (b). Nearly all cells were positive for EBV-EBER by ISH, although intensity of the staining varied from cell to cell (c). Electron microscopy revealed spindle cells with abundant cytoplasmic aggregates of actin microfilaments, including fusiform dense bodies and attachment plaques, confirming the diagnosis of a well-differentiated smooth muscle proliferation (d).

*Patient* 3. The patient is a 25-year-old woman with left kidney agenesis, history of psoriasis since childhood, and seven episodes of shingles. In August 2004, she began intravenous etanercept. One month later, she developed left cervical lymphadenopathy and right lower extremity numbness and tingling. A positive monospot test led to a diagnosis of mononucleosis. Etanercept was discontinued; her symptoms resolved. In October, she began prednisone (40 mg daily) treatment for increasing dyspnea attributed to asthma; this was tapered off in January 2005 when she saw her primary care provider and chest radiograph revealed bilateral infiltrates. Chest CT demonstrated infiltrates and three liver lesions. Lung biopsy revealed *Pneumocystis carinii* pneumonia. CT scan (1/05) confirmed three liver lesions, the largest measuring 7 cm with central necrosis. Fine needle aspiration of a liver mass revealed a well-differentiated SMN. CT scan (4/05) noted that the largest liver lesion was 7.5 cm in size. Subsequent CT scan (8/05) revealed no change. Positron emission tomography (PET) scan (9/05) showed tracer uptake in the liver lesions (Figure 1). In October 2005, she underwent an extended right hepatectomy and cholecystectomy. Pathology revealed multicentric EBV-associated SMNs (Figure 2). She remains without disease as of October 2008.

## 2. METHODS

### 2.1. Light microscopy and immunohistochemistry (IHC)

Formalin-fixed, paraffin-embedded tissue blocks were sectioned (4 *μ*m) and stained with hematoxylin and eosin for conventional histology. IHC analysis was performed using standard techniques with the following antibodies (prediluted from Ventana medical Systems Inc, Tucson, AZ, USA, except where noted): HHF-35, smooth muscle actin (SMA), desmin (DAKO, 1:50), CD117 (DAKO, 1:500), S100 protein (1:500, Dako, Glostrup, Denmark), and HMB-45.

### 2.2. In situ hybridization for EBER

Detection of EBV-encoded early RNA transcripts (EBER 1 and 2) was performed using biotin-conjugated oligonucleotides (PNA probe, Dako), using standard protocols according to the manufacturer’s instructions. The positive control used was a posttransplant EBV-associated diffuse large cell lymphoma. All steps were performed under RNAse-free conditions.

### 2.3. Electron microscopy (EM)

Tissue for EM was available in Case 3. Representative fresh tumor was fixed in 3% formaldehyde—3% glutaraldehyde, postfixed in 1% osmium tetroxide, embedded in epoxy resin, and stained with uranyl acetate-lead citrate using standard procedures. The thin sections were examined using a Philips-410 electron microscope.

## 3. RESULTS

### 3.1. Microscopic and electron microscopic findings

All patient samples were positive for EBV-EBER by ISH (Figure 2), and the vast majority of cells were positive for EBER by ISH. IHC from biopsies of the liver tumor from Patient 1 was positive for SMA and desmin, but negative for CD117 (ckit), HMB-45, and S-100. Histologic features from the colonic SMN from Patient 2 included mild to moderate increase in cellularity, low mitotic count (<1/10 HPF), and no areas of necrosis. IHC was positive for desmin and SMA, but negative for CD117 and S-100.

The liver specimen from Patient 3 revealed multicentric EBV-associated SMNs consisting of four nodules ranging in size from 0.4 cm to 6.7 cm (Figure 2). Moderate increased cellularity (with areas of somewhat rounder, less fusiform cells), focal areas of necrosis, lack of nuclear pleomorphism, and low mitotic rate (1 mitotic figure/50 high-powered field) were noted. The tumors were positive for SMA, HHF35, desmin and negative for CD117. EM from Patient 3 revealed spindle cells with abundant cytoplasmic aggregates of actin microfilaments, including fusiform dense bodies and attachment plaques (Figure 2). Linear arrangements of pinocytotic vesicles were noted in many cells. Cytoplasmic organelles were mainly represented by mitochondria, while rough endoplasmic reticulum cisternae were sparse. These ultrastructural features confirmed the diagnosis of a well-differentiated SMN. No nuclear viral particles were detected.

## 4. DISCUSSION

Multiple lines of evidence implicate EBV in the formation of SMNs [4, 8]. EBV DNA and RNA have been demonstrated in SMNs in immunocompromised patients [4, 5] and in our patients. Conversely, EBV is absent in sporadic smooth muscle tumors in immunocompetent patients [7, 11]. Pathogenesis appears to be related to infection and transformation of smooth muscle cells by EBV; fusion of EBV-superinfected lymphoblastoid cells and human embryonic fibroblasts may be the vehicle by which smooth muscle cells are infected [12]. CD21 expression may play a role, in pathogenesis, as CD21 has been described in 16 of 20 EBV-associated SMNs in patients with AIDS [4, 6, 9]. Moreover, five to ten percent of smooth muscle cells display strong immunofluorescent staining of CD21 [13]. Based on viral genomic analyses of tumors, multifocal lesions appear to result from multiple independent infection events rather than metastasis [14]. It appears that the virus is not overtly virulent, since EBV seems to effect change in smooth muscle cells of immunocompromised patients rather than immunocompetent patients. Perhaps this is why these tumors tend to be low grade.

Histologically, EBV-associated SMNs have a mild to moderate increase in cellularity, low mitotic count (<1/10 HPF), and little to no areas of necrosis. Others have reported foci of primitive-rounded cells and intratumoral T lymphocytes [14, 15], but these were not seen in our three cases. Immunohistochemically, SMNs stain positively for desmin and SMA. On EM, SMNs demonstrate spindle cells with abundant cytoplasmic actin microfilaments with linearly arranged pinocytic vesicles. SMNs appear to be relatively well differentiated with only a modest degree of atypia [14].

In a literature review of 19 reports of EBV-associated SMNs following organ transplantation [16], SMNs occurred after a latency of one to six years as a single tumor, or more often, as multifocal or multicentric lesions in multiple organs [16]. The most common locations were liver, lung, heart, and colon [16, 16–18]. As the behavior of SMNs appears related to immune status rather than to specific histologic features, the mainstay of treatment was the reduction of immunosuppression; however, five of the seven patients who survived with follow-up of 10 months to 12 years underwent surgical resection [16].

Immunosuppression predisposes patients to the development of SMNs, first described 40 years ago [19]. Initially reported, following renal transplantation and immunosuppression in the pediatric population, SMNs are observed in patients after solid organ and stem cell transplants [2–5], with AIDS [6], or with congenital immunodeficiency [7–10]. Reversal of immunosuppression, effective in treatment of posttransplant lymphoproliferative disorder, appears critical to improved outcomes of EBV-associated SMNs [18] (e.g., reduction or change of immunosuppressive agent in transplant patients or use of famciclovir for high-risk patients [17]).

In our case series, the three patients with SMNs were immunodeficient for different reasons (Table 1). We describe the first EBV-associated SMN in a human patient receiving etanercept, although immune dysregulation associated with her psoriasis was more likely an important predisposing condition. Etanercept competitively binds TNF to reduce TNF activity from excessive inflammatory levels to normal physiologic levels, effectively treating rheumatoid arthritis and psoriasis within months [20]. Known side effects of etanercept include an increased risk of lymphoma in these patients [20–22], although it has been reported that long-term treatment with TNF-alpha inhibitors does not increase EBV load in patients with rheumatoid arthritis [23]. Psoriasis patients treated with etanercept have also been diagnosed with noncutaneous solid tumors, nonmelanoma skin cancer, and melanoma as well as an increased risk of liver cancer [24, 25]. Anti-TNF-alpha treatment has been associated with vascular smooth muscle cell proliferation in a rabbit model [26].

In summary, these cases highlight the variable aggressiveness and clinical outcomes of SMNs in patients with immunosuppression on the basis of autoimmunity, iatrogenic immunosuppression, and that caused by HIV. Chemotherapy and surgery both play a role in patients with these usually indolent diseases. It remains to be seen whether effects on EBV itself with antiviral agents or with EBV-specific T cell therapy [27] might have salutary effects against this rare soft tissue neoplasm.

## Figures and Tables

**Table 1:** Three different clinical scenarios in which EBV-related SMNs developed: patient summary. Dx = diagnosis. SMN = smooth muscle neoplasm. DOD = dead of disease. AWD = alive with disease. NED = no evidence of disease.

#	Age at Dx (year)	Cause of immune dysregulation	Date of start of immune dysregulation	Time to SMN	Site	Treatmen	Status follow-up
1	55M	Cadaveric renal transplant prednisone, cyclosporine, azathioprine, mycophenolate mofetil	1994	8 years	Liver (caudate 14 cm); lung	Observation (declined systemic therapy) (7/02)	DOD 5 months

2	18F	AIDS CD4 count = 8	Birth	11 years	Colon, chest, paraspinal/ vertebral, liver, gluteal, perigastric areas	Antiretrovirals doxorubicin, then dacarbazine (10/03)	AWD without progression, 5 years

3	25F	Psoriasis, etanercept therapy	20+ years, 8/04	20+ years, 5 months after etanercept started	Liver	Stopped immunosuppressives; hepatic resection (10/05)	NED, 3 years
